# Sex dimorphism and tissue specificity of gene expression changes in aging mice

**DOI:** 10.1186/s13293-024-00666-4

**Published:** 2024-10-31

**Authors:** Dantong Zhu, Matt Arnold, Brady A. Samuelson, Judy Z. Wu, Amber Mueller, David A. Sinclair, Alice E. Kane

**Affiliations:** 1 Institute for Systems Biology, Seattle, WA 98109 USA; 2 Department of Genetics, Paul F. Glenn Center for Biology of Aging Research at Harvard Medical School, Blavatnik Institute, Boston, MA 02115 USA; 3 Cell Press, Cambridge, MA 02139 USA; 4 Department of Laboratory Medicine and Pathology, University of Washington, Seattle, WA 98115 USA

**Keywords:** Mice, Sex dimorphism, Aging, Tissue-specific, Gene expression, Feature selection, Co-expression network analysis, Muscle, Liver, Adipose tissue

## Abstract

**Background:**

Aging is a complex process that involves all tissues in an organism and shows sex dimorphism. While transcriptional changes in aging have been well characterized, the majority of studies have focused on a single sex and sex differences in gene expression in aging are poorly understood. In this study, we explore sex dimorphism in gene expression in aging mice across three tissues.

**Methods:**

We collected gastrocnemius muscle, liver and white adipose tissue from young (6 months, n = 14) and old (24 months, n = 14) female and male C57BL/6NIA mice and performed RNA-seq. To investigate sex dimorphism in aging, we considered two levels of comparisons: (a) differentially expressed genes between females and males in the old age group and (b) comparisons between females and males across the aging process. We utilized differential expression analysis and gene feature selection to investigate candidate genes. Gene set enrichment analysis was performed to identify candidate molecular pathways. Furthermore, we performed a co-expression network analysis and chose the gene module(s) associated with aging independent of sex or tissue-type.

**Results:**

We identified both tissue-specific and tissue-independent genes associated with sex dimorphism in aged mice. Unique differentially expressed genes between old males and females across tissues were mainly enriched for pathways related to specific tissue function. We found similar results when exploring sex differences in the aging process, with the exception that in the liver genes enriched for lipid metabolism and digestive system were identified in both females and males. Combining enriched pathways across analyses, we identified amino acid metabolism, digestive system, and lipid metabolism as the core mechanisms of sex dimorphism in aging. Although the vast majority of age-related genes were sex and tissue specific, we identified 127 hub genes contributing to aging independent of sex and tissue that were enriched for the immune system and signal transduction.

**Conclusions:**

There are clear sex differences in gene expression in aging across liver, muscle and white adipose. Core pathways, including amino acid metabolism, digestive system and lipid metabolism, contribute to sex differences in aging.

**Supplementary Information:**

The online version contains supplementary material available at 10.1186/s13293-024-00666-4.

## Plain Language Summary

Aging is a complex process that occurs differently across tissues, and in men compared to women. However, the mechanisms that cause sex differences are not well understood. Using naturally aging mouse models we compared how specific genes were differently expressed in muscle, liver and fat of old and young female and male mice. We found that the vast majority of genes that were changed with age were only changed in one sex and specific tissues. Overall, sex differences in aging across tissues were related to genes involved in amino acid metabolism, digestive system and lipid metabolism. Notably, lipid metabolism is important in aging females across all tissues. We also identified a set of genes associated with aging independent of sex and tissue-type involved in immune pathways and signaling. These results enhance our understanding of sex differences in aging.

**Supplementary Information:**

The online version contains supplementary material available at 10.1186/s13293-024-00666-4.

## Highlights

- We performed RNA-seq in three tissues from young (6 months) and old (24 months) mice of both sexes, and applied differential expression analysis and machine learning approaches to identify candidate genes to decipher sex dimorphism in aging.
- We demonstrated that the vast majority of age-related genes were sex and tissue specific, with liver showing greater changes than muscle or white adipose tissue. Enriched pathways underpinning sex differences for each tissue aligned with the function of the tissues.
- In addition to tissue-specific findings, we also observed pathways in amino acid metabolism, digestive system and lipid metabolism contributing to tissue-wide sex dimorphism.
- We applied an unsupervised machine learning approach to select key genes involved in aging regardless of sex and tissue, and these genes were related to the immune system and signaling transduction.

**Supplementary Information:**

The online version contains supplementary material available at 10.1186/s13293-024-00666-4.

## Background

Aging is a gradual and continuous process that involves a recognizable decrease in physiological capacity and response to environmental stresses [1, 2]. Age is also the greatest risk factor for many chronic diseases, including coronary artery disease, diabetes, and Alzheimer’s disease [3, 4].

Recent work has suggested twelve molecular, cellular, and systemic hallmarks of aging [5], providing a point-of-entry for understanding aging. However, the exact mechanisms of how these hallmarks arise still remains unclear. Many of these defined hallmarks, specifically epigenetic alterations, genomic instability and splicing dysregulation result in gene expression changes in aging [6, 7]. Notably, stress response pathways, inflammation and immune responses are among the most common pathways that are upregulated in aging across multiple species [6, 8–10]. In contrast, metabolism and mitochondrial function are generally downregulated [11–13]. Despite some unifying gene expression hallmarks, many of the transcriptional features of aging are tissue and/or cell type dependent [14, 15]. For example, liver is found to show substantial transcriptional change during the aging process compared to other tissues, especially enriched in immune response and metabolic processes [16, 17].

Sex dimorphisms in aging are widely observed across many levels. Most notably, on average women live longer than men, however, women are more frail than men [18]. There are also clear sex differences in the risk and prevalence of age-related diseases [19, 20]. Recently, efforts have been made to explore the underlying mechanisms of these sex differences in aging [21]. For instance, a large-scale UK Biobank study found that mitochondrial abundance is elevated in women compared to men, and is negatively related to advanced age [22]. Sex differences are also observed in cellular senescence in adipose tissue [23], and mTOR signaling in multiple tissues [24]. Through gene expression analysis, several studies have shown that regulation of xenobiotic metabolism related genes (e.g. cytochrome P450 genes) in the liver are sex dependent during aging in multiple mammalian animals [25–27]. Sex differences in gene expression with age are also observed in human muscle, where replicative senescence and cytochrome C release are disrupted in aged male but not female skeletal muscle [28]. However, the vast majority of aging gene expression studies use only a single sex, usually males, and there is little known about sex differences in transcriptional changes in aging.

Gastrocnemius muscle (skeletal muscle, hereafter Muscle), liver (hereafter Liver) and white adipose tissue (hereafter Wat) present massive changes during the aging process, and have been extensively studied on the gene expression dynamics in aging. In this study, in order to elucidate sex differences in tissue aging, we measure gene expression in Muscle, Liver and Wat from young and old C57BL/6NIA mice of both sexes. First we explore differentially expressed (DE) genes in old females compared to males, and find both tissue-specific and tissue-independent genes associated with sex dimorphism in aged mice. Secondly, we looked at genes that were changed with age (between young and old groups) across both sexes. Using both DE analysis and feature selection, we found that the majority of genes changed in aging were both sex and tissue specific. The key pathways underpinning sex dimorphism in aging were enriched for amino acid metabolism, digestive system and lipid metabolism. Notably, apart from sex differences, age-related genes in females were enriched for lipid metabolism across tissues. We also used an unsupervised machine learning approach, co-expression network analysis, to identify a set of core genes, enriched for immune and signaling pathways, that were changed in age independent of sex or tissue-type. We dig deeper into both tissue-specific and tissue-independent genes associated with aging. By looking at not only the differences but also the similarities of these 3 tissues, our results provide insights into the mechanisms underpinning the sex dimorphism and tissue specificity of the aging transcriptome.

## Methods

### Mice

C57BL/6NIA mice (male and female) were obtained from the National Institute on Aging (NIA) Aging Rodent Colony. Mice were group housed (3–4 mice per cage) at Harvard Medical School in ventilated microisolator cages with a 12 h light cycle (6 am–6 pm), at 71°F with 45–50% humidity and fed AIN-93G Purified Rodent Diet (Dyets Inc, PA). All animal experiments were approved by the Institutional Animal Care and Use Committee of the Harvard Medical Area.

### Tissue sample collection

**Fig. 1:**
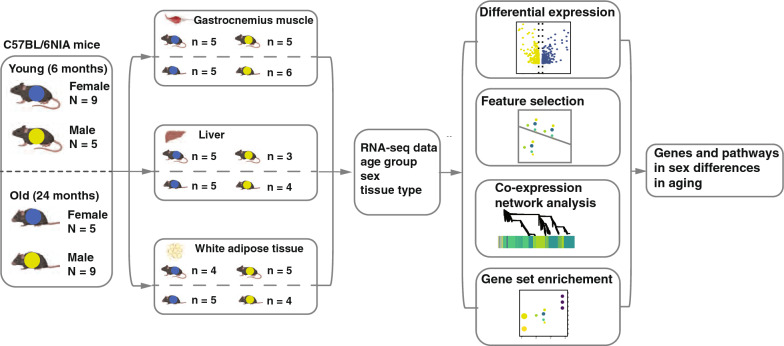
Schematic figure showing the workflow of the study. Blue dots represent female samples and yellow dots represent male samples

For tissue collections (between 10am and 1 pm), mice were given a terminal dose of sodium pentobarbital intraperitoneally, and full anesthesia confirmed. The abdominal cavity was opened, the mouse exsanguinated, and then gastrocnemius muscle, liver, and abdominal white adipose tissue collected and flash frozen in liquid nitrogen. Tissues were collected from mice at two age groups [old, 24 months, N = 14 (Female, N = 5 and Male, N = 9); young, 6 months, N = 14 (Female, N = 9 and Male, N = 5)] (Fig. 1).

### RNA sequencing and raw data processing

These data are control samples from a larger intervention study, so detailed methods can be found in Kane et al. [29]. Briefly, 30–40 mg of frozen tissue was pulverized and RNA extracted using a Trizol-chloroform protocol, and cleaned using the NucleoSpin RNA kit (Macherey–Nagel). RNA quality and concentration was determined using a 4200 Tapestation System (Agilent), and only samples with a RIN score > 7.5 were used for further analysis. In total, there were 56 samples (gastrocnemius muscle, n = 21; liver, n = 17; and white adipose tissues, n = 18) subjected to RNA sequencing and ensuing bioinformatics analyses. mRNA library preparations and sequencing were done by Novogene using a Poly A enrichment-based library prep, and a Novoseq PE150 kit for sequencing. Sequencing quality was checked with FastQC (v. 0.12.0), and reads mapped with hisat2 (v. 2.2.1) (samtools, v. 1.19) using the mm10 (UCSC) reference genome. Resulting sam files were used to make raw feature counts matrices.

### Differentially expressed genes (DEG) analysis

Gene counts data were subjected to DE analysis using the ‘limma’ (v. 3.56.2) pipeline [30]. Briefly, genes that had maximum counts per million (cpm) values less than 10 across all 56 samples were labeled as unexpressed genes and were excluded from the ensuing analysis, with 18,364 genes remaining. Gene counts for each sample were then log transformed and the mean–variance relationship in the data were estimated, with a weight assigned to each observation (voom method) [31]. Design matrix for differential expression analysis, including age, sex and tissue was determined by principal component analysis (Additional File 1). The data with weight information along with the multi-factor design matrix were then subjected to linear modeling with empirical bayes smoothing of gene-wise standard deviations. For comparisons between two conditions, for instance female vs. male samples in the old age group, genes that had an absolute Fold Change (FC) greater than log_2_(1.5) determined by t-tests relative to a threshold (TREAT) method [32] (adjusted p-value by the Benjamini–Hochberg method less than 0.05) were defined as DEGs.

### Feature selection by elastic net

We performed feature selection in three subsets of samples, (a) female and male samples from the old age group; (b) old and young age group from the female samples; and (c) old and young age group from the male samples. Within each subset, we fit a logistic regression model using the log-transformed and normalized gene expression data derived from limma-voom pipeline as the independent variables (all gene features, n = 18,364; non sex chromosome related gene features, n = 15,932) and the condition (old vs. young groups or female vs. male groups) as the dependent variable. We applied a 20 × repeated fivefold cross validation approach for the feature selection. Briefly, we performed 20 runs of multivariate logistic regression with elastic net regularization. Within each run, the hyperparameters were tuned using fivefold cross-validation, and a list of gene features assigned a non-zero coefficient was derived. These lists were combined into a list of gene features, which were then ranked according to the importance of the individual gene, i.e. absolute average coefficient over 20 runs. For each subset, we selected the top 20 features with the highest importance as features for the subset.

### Co-expression network analysis

To cluster genes that have similar expression patterns across 56 samples, we performed weighted correlation network analysis using the WGCNA package (v. 1.72-5) [33]. As clear sex differences have been revealed by DEG analysis, we only included somatic chromosome related genes (n = 15,932) in a single block with soft thresholding at 6. For each gene cluster/module determined, the 1st principal component was derived as the module eigenvalue. We then fit a logistic regression model using the eigenvalue as the independent variable, and age group as the dependent variable, and further adjusted for sex and tissue. The module(s) that reached a significant (0.05 divided by the total number of modules) level of association with the binary age group variable regardless of sex and tissue, was selected as the candidate module(s). Within the module, genes that showed gene significance greater than 0.2 (correlation coefficient between the gene expressions and the binary age group variable) and module membership greater than 0.8 were selected as the hub genes in the module.

### Gene set enrichment analysis

Enrichment analysis was performed across gene sets derived from differential expression analysis, gene features selected by elastic net, and hub genes within the candidate gene module. Each gene set was subjected to gene set enrichment analysis by Enrichr (v. 3. 2) [34] to select the Kyoto Encyclopedia of Genes and Genomes (KEGG) pathways that were overrepresented. The overrepresentation of KEGG pathways was tested by Fisher’s exact test and p-value was adjusted by Benjamini–Hochberg method. KEGG pathways that presented adjusted p-values that were less than 0.05 were considered as enriched pathways. For each pathway, gene ratio was calculated by using the number of genes identified within the pathway divided by the total number of genes within the pathway.

## Results

### Clustering of samples by principal component analysis

To observe clusters and potential outliers in the data, we performed a principal component analysis (PCA) on the gene expression levels of the selected 18,364 genes across all samples (Additional File 1). The PCA plot suggests clear separation of samples by principal component (PC)1 and PC2, forming three clear clusters for Muscle, Liver and Wat, respectively. Within each tissue cluster, the separation of samples across age and sex groups can also be observed.

### DEGs across females and males in the old age group

**Fig. 2:**
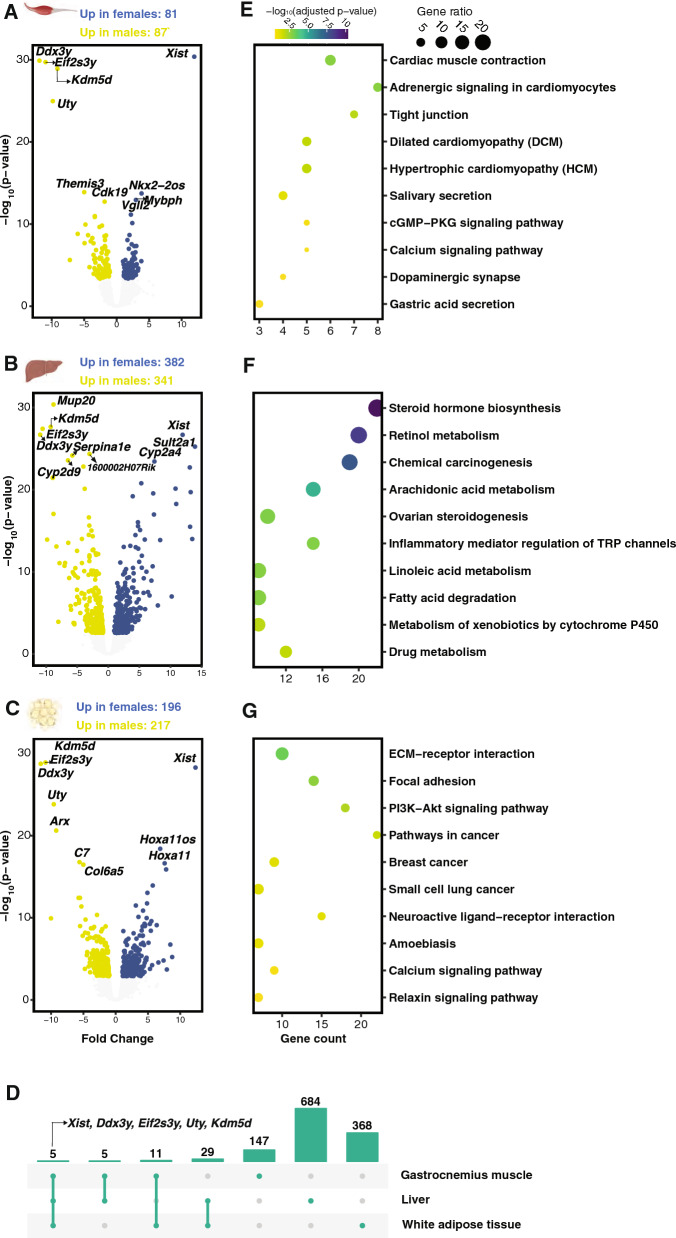
Differential expression analysis results for males and females in the old age group. The differential analysis was performed by using the limma-voom pipeline, which included tissue, sex and age group as factors in the design. Differentially expressed genes between females and males in the old age group were defined as genes that present absolute Fold Change greater than log_2_(1.5) by t-test relative to a threshold with adjusted p-value less than 0.05. Volcano plots show the up- (blue) and down- (green) regulated genes when comparing female to male samples in three types of tissues, gastrocnemius muscle (**A**), liver (**B**), and white adipose tissue (**C**). Genes that were not determined as DEGs are colored in gray. By combining three sets of DEGs, we generated an Upset plot (**D**) showing intersecting sets, with five genes shared across the three DEG sets. Unique DEG sets were subjected to gene set enrichment analysis, and top ten pathways that were overrepresented and showed the least adjusted p-values were plotted for gastrocnemius muscle (**E**), liver (**F**), and white adipose tissue (**G**), respectively. The position of the dot at the x-axis shows the number of genes within the unique DEG set, color indicates the significance level and size represents the gene ratio in the complete gene set of the pathway

To determine differentially expressed genes (DEGs) between older females and older males, we performed global differential gene expression analysis, followed by contrasts of females and males within each individual tissue of the old group only. The results revealed 168, 723, and 413 genes that were differentially expressed in Muscle, Liver and Wat respectively (Fig. 2A–C and Additional File 2). For each tissue there were relatively equal amounts of genes that were either up or down regulated in old females relative to old males. Interestingly, only five genes were differentially expressed across all three tissues (Fig. 2D): *Xist*, *Ddx3y, Eif2s3y*, *Uty*, and *Kdm5d,* and these are all sex-chromosome linked genes. The vast majority of DEGs in each tissue were not shared, suggesting tissue specific gene regulation mechanisms between older females and males. The unique DEG set in each tissue, 147 genes (87.5% of Muscle DEGs), 684 genes (94.6% for Liver DEGs) and 368 genes (89.1% for Wat DEGs), were respectively subjected to gene set enrichment analysis. Pathway enrichment results revealed distinct pathways that underpin the sex differences (Fig. 2E–G) in the three tissues. For Muscle, the majority of enriched pathways were involved in the circulatory system or cardiovascular diseases, which were up-regulated in females compared to males (Additional File 3A). For Liver, DEGs were predominantly related to the digestive system and lipid metabolism, wherein pathways were mainly up-regulated in females (Additional File 3B). Meanwhile, we observed that down-regulated genes (in Liver DEGs comparing females to males) were enriched for amino acid metabolism, lipid metabolism and immune system (Additional File 3C). For Wat, pathways were predominantly related to signal transduction and these pathways were down-regulated in females (Additional File 3D), with only one pathway, neuroactive ligand-receptor interaction, found up-regulated in females.

### Gene features predictive of sex in the old age group

After determining tissue-specific DEGs from the comparisons between females and males in the old age group, we then deployed a generalized linear model with elastic net regularization to select gene features predictive of sex, regardless of tissue-type, in the older group. We excluded sex-chromosome linked genes from this analysis. Out of 15,932 somatic chromosome related genes, we identified the top gene features that are predictive of female sex in the old age group (Additional File 4A). Gene expression levels of 16 genes (including *Smarcc1, Kansl1* and *Taf2)* were positively associated with female sex independent of tissue type and 4 genes were negatively associated. Enrichment analysis identified no candidate pathways across these genes, and none of the genes overlapped with those identified in the DE analysis above (Fig. 2).

### Sex differences in DEGs of aging

**Fig. 3:**
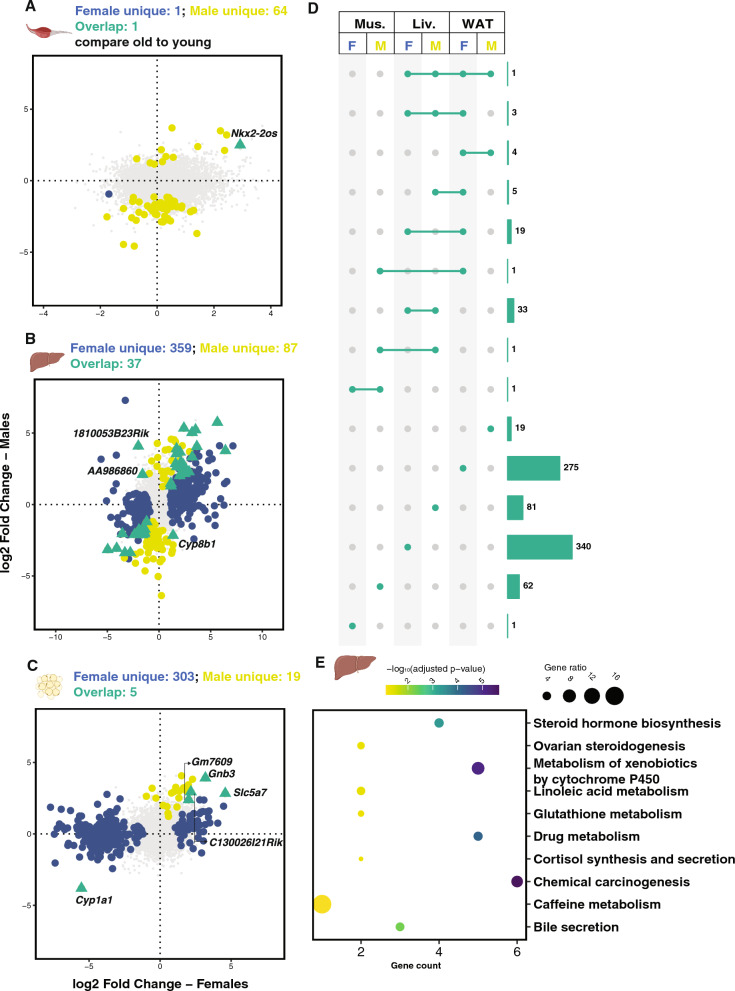
Sex differences in aging in terms of differentially expressed genes (DEGs). The differential analysis was performed by using the limma-voom pipeline, which included tissues, sex and age group as factors in the design. DEGs under two conditions (comparisons of the old to the young group in either males or females) were defined as genes that present absolute Fold Change greater than log_2_(1.5) by t-test relative to a threshold with adjusted p-value less than 0.05. The scatter plots show logFCs of all the genes in female and male comparison pairs. DEGs that were unique in females are colored in blue, with yellow for unique male DEGs and green triangle for common DEGs, in three types of tissues, gastrocnemius muscle (**A**), liver (**B**), and white adipose tissue (**C**). Genes that were not determined as DEGs are colored in gray. By combining the above six sets of DEGs, we generated an Upset plot (**D**) showing intersecting sets. **E** The shared DEGs across males and females for liver (n = 37) were subjected to gene set enrichment analysis, and top ten pathways that were overrepresented and showed the least adjusted p-values were plotted. The position of the dot at the x-axis shows the number of genes within the DEG set, color indicates the significance level and size represents the gene ratio in the complete gene set of the pathway

Following the investigation of sex differences in DEGs for the old age group, we analyzed sex differences in the aging process, i.e. comparing the old with the young age groups. First, we compared the old and young age groups in each sex and tissue group separately. The analyses revealed 2 DEGs in females and 65 in males for Muscle (Fig. 3A). There were only one gene, *Nkx2-2os*, differentially expressed in both sexes. In Liver, 396 DEGs for females and 124 for males were detected with 37 genes shared (Fig. 3B). 34 shared genes showed a consistent direction of changes in both males and females while, notably, there were three genes, *1810053B23Rik*, *AA9866860*, and *Cyp8b1* with opposite direction of changes across sexes, suggesting distinct regulation mechanisms of these genes during the aging process in females and males. All together, enrichment analysis of the shared DEGs across sexes in the liver identified lipid metabolism pathways, including retinol metabolism and steroid hormone biosynthesis (Fig. 3E). In Wat, there were 308 and 24 DEGs in females and males respectively and 5 genes overlapped (Fig. 3C).

We combined the 6 DEG sets derived from above to determine if there were overlapping genes, but found the majority of the DEGs were tissue and sex specific (Fig. 3D). Enrichment analysis was undertaken on the unique DEGs with age (old vs young) in each sex and tissue group that had at least 30 genes (Additional File 5). For Muscle in males, enriched pathways changed in age included signaling transduction, and for Wat in females, pathways were focused on the digestive system. Despite the enrichment sets including sex specific genes only, for Liver, both males and females were enriched for lipid metabolism.

### Global gene expression features in aging in each sex

Following the tissue specific DE analysis in the aging process, we again used generalized linear regression with elastic net regularization to select gene features predictive for age in either females or males, regardless of tissue. We used gene expression data from 18,364 genes, including both sex-chromosome and somatic chromosome linked genes. We derived the top 20 gene features for age within the female samples, with 1 positively predictive for the old age group, and the rest negatively associated (Additional File 4B). For the 20 gene features identified for the male samples, 11 gene features were positively predictive for the old age group (Additional File 4C). No gene features were shared across the two sets. Gene features from female samples were enriched in mRNA surveillance pathways, and features from male samples were mainly enriched in environmental adaptation.

### Overall pathways driving sex differences in aging

**Fig. 4:**
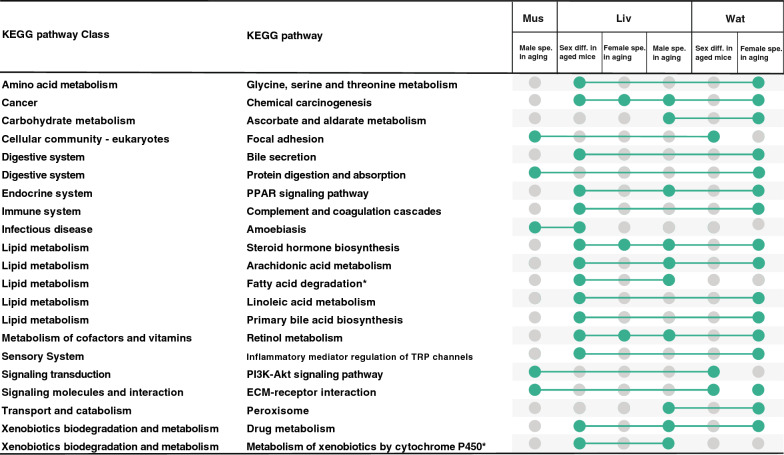
List of 21 enriched KEGG pathways from identified differentially expressed gene (DEG) lists. DEG lists were derived from differential expression analysis in gastrocnemius muscle (Mus), liver (Liv), and white adipose tissue (Wat) from either comparing females to males in the old age group (labeled Sex diff. in aged mice) or unique sex specific genes in aging (comparing the old to young age group) (labeled Female or Male spe. in aging). Enriched KEGG pathways were determined by the overrepresentation of genes by Fisher’s exact test, where adjusted p-values were calculated using the Benjamini–Hochberg method and cutoff at 0.05 was applied. KEGG pathways that were detected in more than one gene set were obtained. The green dots represent the presence of the pathway within the enrichment analysis of the gene list. *The pathway was only detected in one type of tissue

In order to understand if tissue-specific gene sets were enriched for universal pathways which may drive sex differences in aging, we derived a total of 89 enriched pathways from 7 DEG lists, i.e. 3 DEG lists from two sexes comparison of the old group (one per tissue) and 4 sex specific DEG lists from age group comparisons (for those comparisons with > 30 genes, male Muscle, female Wat and both sexes Liver). From the 59 unique pathways, we selected 21 pathways which were present in more than one DEG list, and 19 pathways which were present in more than one tissue (Fig. 4). The resulting pathways were within classes such as amino acid metabolism, digestive system and lipid metabolism. Apart from the sex differences, when considering the two female specific DEG lists (female specific Wat and Liver) simultaneously, lipid metabolism pathways were detected enriched across tissues.

### Gene module associated with aging

**Fig. 5:**
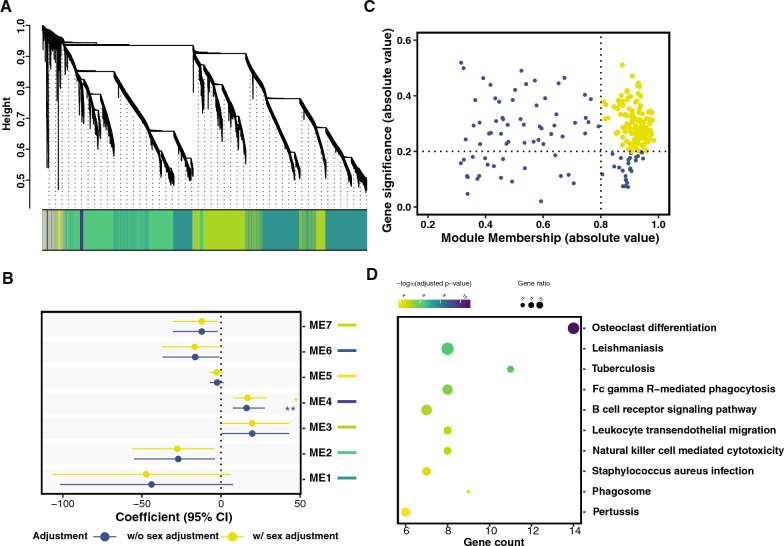
Gene module associated with aging. Gene co-expression network analysis was performed based on gene expression levels for somatic chromosome-related genes (n = 15,932) from all 56 samples, regardless of tissue and sex. The analysis resulted in 7 gene modules (**A**). Within each module, a co-expression network was derived and the first principal component of gene expression was denoted as module eigenvalue. **B** Forest plot shows the associations of eigenvalue of each module with age group and ME4 presented significant association with age group regardless of sex. Based on gene significance (correlation coefficient between gene expression level and age group) and module membership, hub genes were selected (**C**). These hub genes were then subjected to gene set enrichment analysis, and top ten pathways that were overrepresented and showed the least adjusted p-values were plotted (**D**). The position of the dot at the x-axis shows the number of genes within the DEG set, color indicates the significance level and size represents the gene ratio in the complete gene set of the pathway

Our previous analysis required comparisons of a pair of conditions. Next, we used an unsupervised machine learning approach to investigate global gene expression features associated with the aging process regardless of tissue and sex. We performed a co-expression network analysis to facilitate the detection of a key gene module. From the gene expression patterns, we identified seven gene modules (ME1 to 7) from somatic chromosome-related genes (Fig. 5A), represented in different colors. ME4, which contains a set of 222 genes, showed significant association between the gene module eigenvalue (PC1) and age group, regardless of sex, suggesting genes within this module are related to aging (Fig. 5B). Based on gene significance and module membership, we identified 127 hub genes in the network (Fig. 5C and Additional File 6) and these genes were enriched in the immune system and signaling transduction (Fig. 5D). 40 of the genes were overlapping with genes identified by the previous DEG analysis, including 36 genes from Liver and 8 from Wat (Additional File 7).

## Discussion

Sex differences in age-related gene expression across tissues are not well understood. Here, using naturally aging mouse models, we compared gene expression across three tissue types, gastrocnemius muscle, liver and white adipose tissue in old (24 month) and young (6 months) females and males. We applied DE analysis and gene feature selection by machine learning approaches to identify sex and tissue specific genes whose expression levels are associated with aging. We found that the vast majority of age-related genes were sex and tissue specific. Overall, sex differences were driven by pathways in amino acid metabolism, digestive system and lipid metabolism, and genes specifically associated with aging in females were enriched for lipid metabolism. Using machine learning approaches, core genes associated with aging independent of sex and tissue-type were also identified, and these were enriched for immune pathways and signaling.

### Tissue-specific sex differences

Tissue type explained the vast majority of variance in our gene expression datasets, more so than sex or age (Additional File 1). This is not surprising as it has been observed in previous studies in humans and mammals in the field of aging [9, 11, 25]. We also observed significant sex dimorphisms when comparing the female and male samples both in the old age group and the age group changes, i.e. the aging process.

The majority of the genes contributing to sex differences were tissue specific, evidenced by extremely limited common genes (Figs. 2D and 3D). Within the old age group, these DEGs were enriched for pathways in the circulatory system for Muscle, lipid metabolism for Liver, and signaling pathways for Wat, which aligns with each tissue’s specialized functions and environment [16]. We looked into the specific regulation pattern of these pathways based on gene expression levels, and found circulatory system pathways (in Muscle) and lipid metabolism and digestive system pathways (in Liver) were up-regulated in females compared to males. Down-regulated genes in females were involved in amino acid metabolism, lipid metabolism and immune system (in Liver) and signal transduction (in Wat).

We observed a slightly different story when looking at the aging process (rather than just comparing within the old age groups). We saw clear sex differences again, with only 1, 37, and 5 genes shared when combining the females and males in three types of tissues, respectively. Interestingly, while more genes changed in Muscle in males, which is also revealed in another study [35], most gene expression changes with age in Liver and Wat were in females. This is possibly due to the fact that loss of muscle mass during aging is more prominent in males [36] and males may have less physiological changes than females in the liver and adipose tissues [37, 38]. For enrichment analysis for sex-specific genes, we observed protein digestion and absorption in male Muscle samples. Protein homeostasis is one of the 12 hallmarks of aging [5], and this result suggests protein metabolism differences in muscle aging of the two sexes. For Wat, we identified female-specific changes in digestive system-related pathways as well as lipid metabolism. Sex differences [39] and aging [40] have been reported to have an impact on adipose tissue, and our results further indicate sex differences in adipose tissue aging.

Liver showed the most overlap (37 genes) between sexes for genes that are changed in aging. Additionally, even when only considering distinct sex-specific gene lists, both male and female genes in Liver were enriched for common pathways including steroid hormone biosynthesis, retinol metabolism and chemical carcinogenesis that is highly related to cytochrome P450 enzymes. In particular, four cytochrome P450 genes, *Cyp2d9*, *Cyp1a2*, *Cyp3a11*, and *Cyp17a1* were similarly changed in aging across males and females. Cytochrome P450 genes have been previously found, either in humans or mice, to be under the influence of age and sex [25, 41]. Lipid metabolism has also been previously shown to change in age and sex [42, 43]. Interestingly, three genes detected in Liver, *1810053B23Rik*, *AA986860*, and *Cyp8b1*, showed opposite directions of change with age in females and males. *Cyp8b1,* another cytochrome P450 gene, increased in females, and decreased in males in our study. A previous study using only male rats observed a decrease in *Cyp8b1* expression between 6 and 24 months [44]*,* and one previous study showed sex differences in *Cyp8b1* levels in young hepatocyte nuclear factor (HNF) 4α knock-out mice [40]. Given the potential role for this enzyme in the treatment of nonalcoholic fatty liver disease and type 2 diabetes [45], more investigation into this intriguing sex difference is needed. *1810053B23Rik*, *AA986860* are currently un-annotated but also present intriguing targets for further investigation. Overall, our aging liver results reveal both sex-dependent and -independent changes and imply key roles of Cytochrome P450 genes and lipid metabolism in aging, across both sexes.

### Sex differences across tissues

Although most changes in aging were tissue specific, we also looked at core genes changed with age between sexes across the three tissues examined. From the differential expression analysis, we found 5 sex chromosome-linked genes (*Xist*, *Ddx3y*, *Eif2s3y*, *Uty*, and *Kdm5d*) were differentially expressed independent of tissue type in the old mice. Intriguingly, these genes have been highly associated with aging or age-related diseases. *Xist* serves as a feature in predicting cellular age [46], and the remaining four genes are related to cardiac diseases in males [47, 48].

We also combined enriched pathways from the DEG lists of sex-specific gene expression changes in aging, and found 19 pathways present in more than one tissue. Among these pathways, we identified three pathway classes, amino acid metabolism, digestive system and lipid metabolism as core pathways changed with age in a sex-specific manner across tissues. Many metabolites related to the above three classes have been found to show sex differences, for instance, glycine and serine [49], bile acid [50] and linoleic acid [51]. Our results further indicate their functions in sex differences in aging. We propose pathways involved in these three candidate classes are potential core mechanisms of sex differences in aging and need further study.

Apart from the merge of DEG analysis results, we utilized a machine learning approach to select gene features associated with sex, independent of tissue type. Of the top 20 genes whose levels were predictive of female sex in the old age group, two interesting genes were *Smarcc1* and *Smarca4*, which encode subunits of SWI/SNF complex, an activity-dependent neuroprotective protein that has previously shown sex dimorphism [52, 53]. For aging, we stratified female and male samples and performed feature selection respectively, and revealed distinct gene sets predictive of aging. We identified mRNA surveillance pathway as an enriched pathway in female samples, wherein gene *PNN* is linked to aging and neurological diseases [54], and revealed thermogenesis and circadian rhythm as enriched pathways in male samples. The latter two pathways have been demonstrated as closely related to aging [55, 56]. These results demonstrate the utility of feature selection using machine learning algorithms, and suggest that, across tissues, males and females have different gene expression profiles in aging.

Taking tissue-specific and tissue-wide analysis together, we have shown strong sex differences in aging. These results highlight the importance of understanding sex differences and provide implications for sex-specific aging interventions.

### General aging

Although we were mostly interested in sex differences, we did explore whether there were core genes associated with aging across both tissues and sexes. Using co-expression network analysis, we selected a set of 127 hub genes from the module that was associated with aging regardless of sex. Of these hub genes, 40 genes overlap with the previously determined genes from DE analysis in Liver and Wat, suggesting key functions of these genes in aging as well as sex differences. We also identified 28 enriched KEGG pathways, wherein 12 pathways were in the immune system class and 2 in the signal transduction class. These results suggested that the immune system and signal transduction are strongly altered during the aging process. Furthermore, as the hub genes were derived from a gene module that was associated with aging regardless of sex and tissue, it can be concluded that these pathways are universally related to aging. These results align with previous findings [57, 58].

### Limitations

There are some limitations to this study. The sample size for RNA sequencing is relatively small, which might decrease the power of statistical analysis. However, C57BL/6NIA mice are genetically identical and present relatively little heterogeneity. Additionally, our study was limited to three tissue types, and it would be interesting in future work to include other tissues, for instance kidney, brain and intestine. Nevertheless, based on the current three types of tissue, we have successfully revealed the tissue specificity of aging. On the other hand, all the mice were kept at the same circadian cycle and tissue samples were collected roughly within 3 h on the same day. It will be interesting to investigate circadian effects on gene expression, although we are not expecting any significant impact leading to biases in the comparisons of the samples. For future work, it would also be ideal to validate these results in other data sets, other mouse strains, and human datasets.

## Conclusion

In summary, we used female and male tissues samples from two age groups of mice to identify candidate gene sets and to investigate the mechanisms involved in sex dimorphism and tissue specificity in aging. The results suggested that sex differences in aging are distinct in different types of tissues. Apart from the diversity, we found four pathway classes, amino acid metabolism, digestive system, lipid metabolism, and xenobiotics biodegradation are the core mechanisms in sex differences in aging.

## Supplementary Information

Additional file 1: Principal components analysis plots of 56 samples based on gene expression data. Samples from three types of tissues were presented in different colors and the condition of samples was indicated by the shape.

Additional file 2: Heatmap plots of gene expression level z-scores. The plots show the z-scores of gene expression levels for genes that were defined as differentially expressed genes under the comparisons between male and female samples in each tissue. There were 168, 723, and 413 genes identified in gastrocnemius muscle, liver and white adipose tissue, respectively.

Additional file 3: Over-represented pathways from tissue specific gene sets in the aged mice. The gene sets were derived from the differentially expressed genes determined when comparing female mice tissue samples to males within the aged group. Unique DEGs not overlapped with other tissues were obtained. For each unique DEG set, genes that had positive logFCs and negative logFCs were subjected to enrichment analysis respectively. Enriched pathways were detected inup-regulated genes in gastrocnemius muscle,up-regulated genes in Liver, down-regulated genes in Liver and down-regulated genes in white adipose tissues.

Additional file 4: Gene features by machine learning approach. (A) Top 20 gene features predictive for sex differences in the old age group, (B) Top 20 gene features predictive for age group in the female samples, and (C) Top 20 gene features predictive for age group in the male samples. The gene features were selected by generalized linear regression with elastic net regularization through a 20-repeated 5-fold cross-validation approach. Top genes features were ranked by the absolute average coefficient over 20 runs. Gene features with positive coefficients were positively predictive for females or the old age group.

Additional file 5: Over-represented pathways from tissue specific gene sets in the aging process. The gene sets were derived from the differentially expressed genes determined when comparing the old to the young age group in three types of tissues and from both sexes respectively. Sex specific DEGs that were not overlapped with the rest of the DEG lists were obtained, and those unique DEG lists containing greater than 30 genes were subjected to enrichment analysis respectively. Enriched pathways were detected in male specific genes in gastrocnemius muscle, female specific genes in Liver, male specific genes in Liver and female specific genes in white adipose tissues.

Additional file 6: Co-expression network by weighted correlation network analysis. Gene expression data from all the 56 samples were subjected to co-expression network analysis. Network of the gene module that was determined associated with aging regardless of sex is shown. Nodes represent candidate hub genes.

Additional file 7: List of overlapping 40 hub genes from the co-expression network analysis. 40 hub genes were also identified in the previously selected differentially expressed genes from either the comparisons between female and male in the old age group or the comparisons between the old and the young age group of both sexes, in liver and white adipose tissue

Additional file 8: Supplementary tables for results of differentially expressed genes, pathway enrichment analysis, and gene modules analysis. *Supplementary table 1*. List of differentially expressed genes in comparing females to males in the old age group; *Supplementary table 2*. List of enriched pathways that are over-presented based on differentially expressed genes detected in each tissue in comparing females to males in the old age group. *Supplementary table 3*. List of enriched pathways that are over-presented based on up- or down- regulated differentially expressed genes detected in each tissue. *Supplementary table 4*. List of differentially expressed genes in comparing old to young age group in females and males respectively. *Supplementary table 5*. List of enriched pathways that are over-presented based on differentially expressed genes detected in each tissue in comparing old to young age group. *Supplementary table 6*. List of genes in gene module ME4 determined by WGCNA. *Supplementary table 7*. Results for association of gene module eigenvalues with age group. *Supplementary table 8*. List of enriched pathways that are over-presented based on hub genes from gene module ME4.

## Data Availability

RNA-seq raw data are deposited to the Sequence Read Archive database under the BioProject accession number PRJNA1172958. Mice metadata, transcript abundance data, and R markdown file for data analysis are available at https://github.com/Kane-Lab-ISB/transcriptome-analysis-in-aging-mice.git. Differentially expressed genes, pathway enrichment analysis, and gene module tables are included in Additional File 8.
