# Supplementary figures and images for: Sex dimorphism and tissue specificity of gene expression changes in aging mice
Source: Biol Sex Differ. 2024 Oct 31;15:89. doi: 10.1186/s13293-024-00666-4 (PMC11529319; doi:10.1186/s13293-024-00666-4)

## Additional file 1

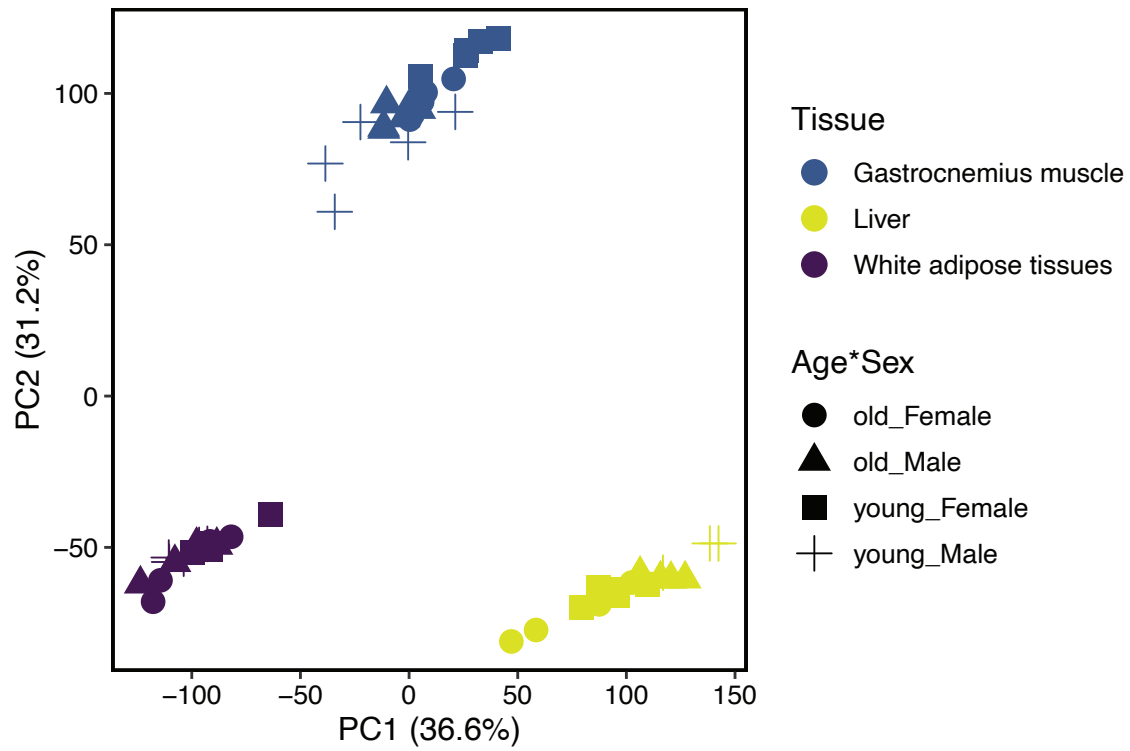

Supplement: Supplementary file 1 — Additional file 1: Principal components analysis plots of 56 samples based on gene expression data. Samples from three types of tissues were presented in different colors and the condition of samples was indicated by the shape. [file 13293_2024_666_MOESM1_ESM.pdf]

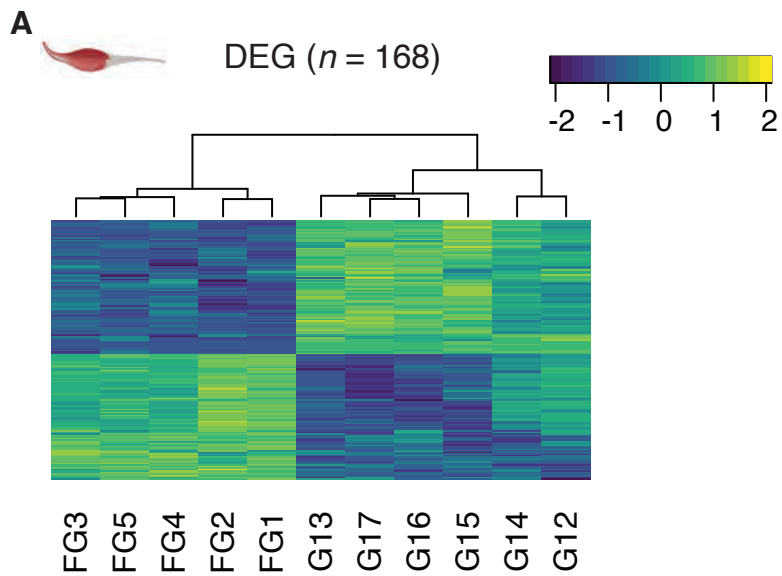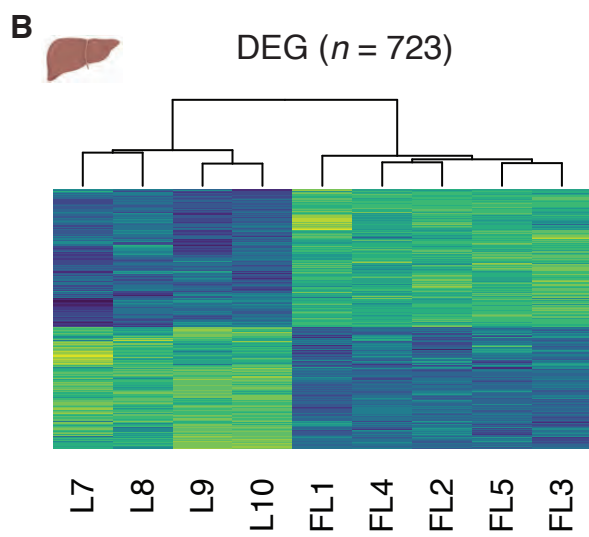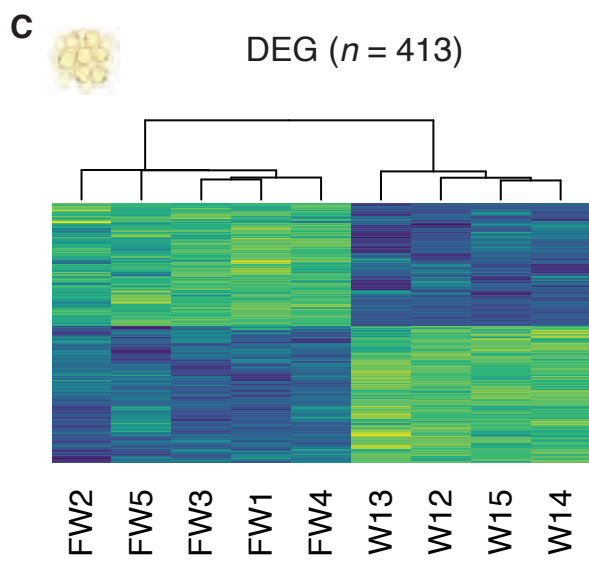

Supplement: Supplementary file 2 — Additional file 2: Heatmap plots of gene expression level z-scores. The plots show the z-scores of gene expression levels for genes that were defined as differentially expressed genes under the comparisons between male and female samples in each tissue. There were 168, 723, and 413 genes identified in gastrocnemius muscle, liver and white adipose tissue, respectively. [file 13293_2024_666_MOESM2_ESM.pdf]

Additional file 3

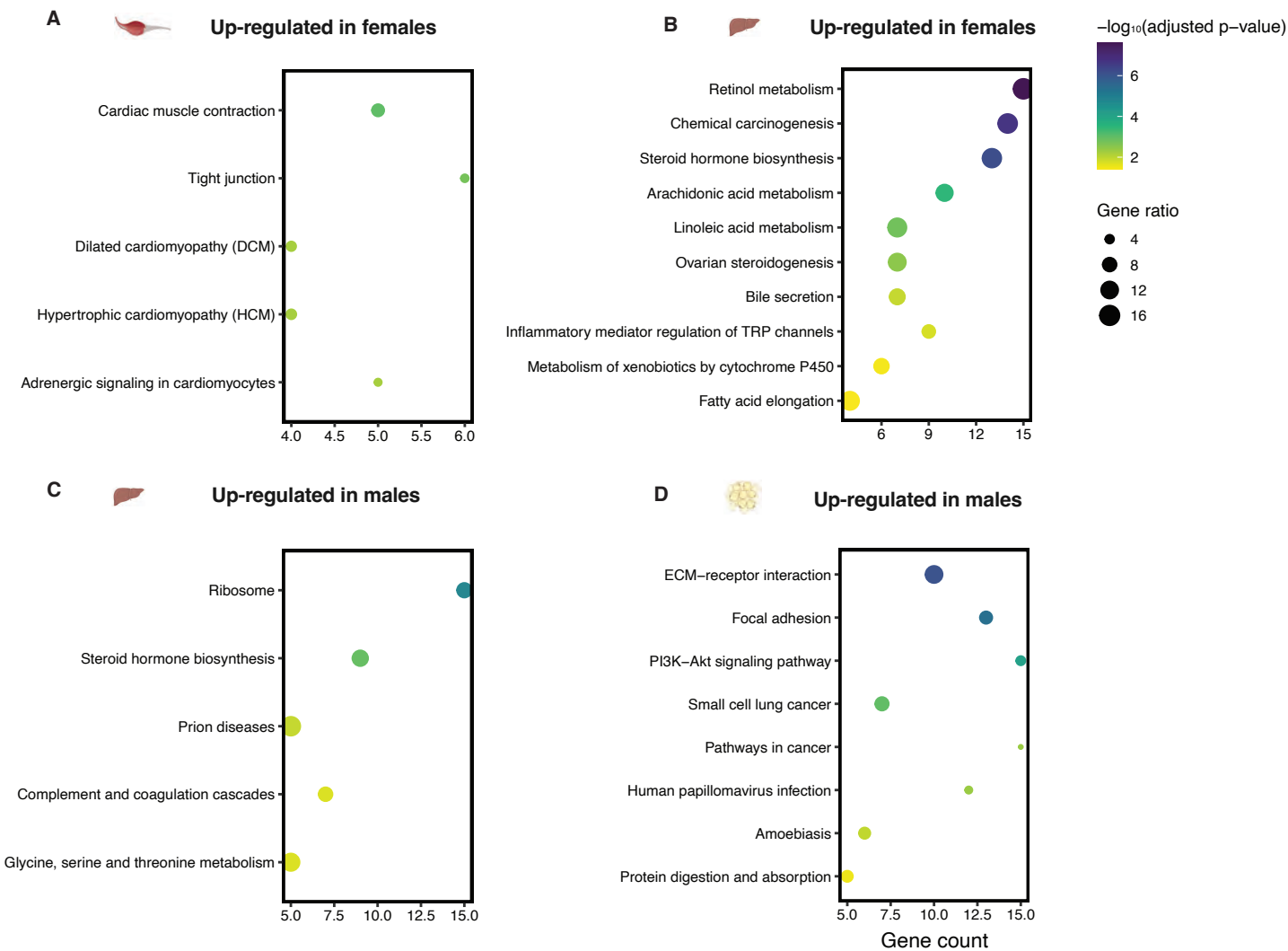

Supplement: Supplementary file 3 — Additional file 3: Over-represented pathways from tissue specific gene sets in the aged mice. The gene sets were derived from the differentially expressed genes determined when comparing female mice tissue samples to males within the aged group. Unique DEGs not overlapped with other tissues were obtained. For each unique DEG set, genes that had positive logFCs and negative logFCs were subjected to enrichment analysis respectively. Enriched pathways were detected inup-regulated genes in gastrocnemius muscle,up-regulated genes in Liver, down-regulated genes in Liver and down-regulated genes in white adipose tissues. [file 13293_2024_666_MOESM3_ESM.pdf]

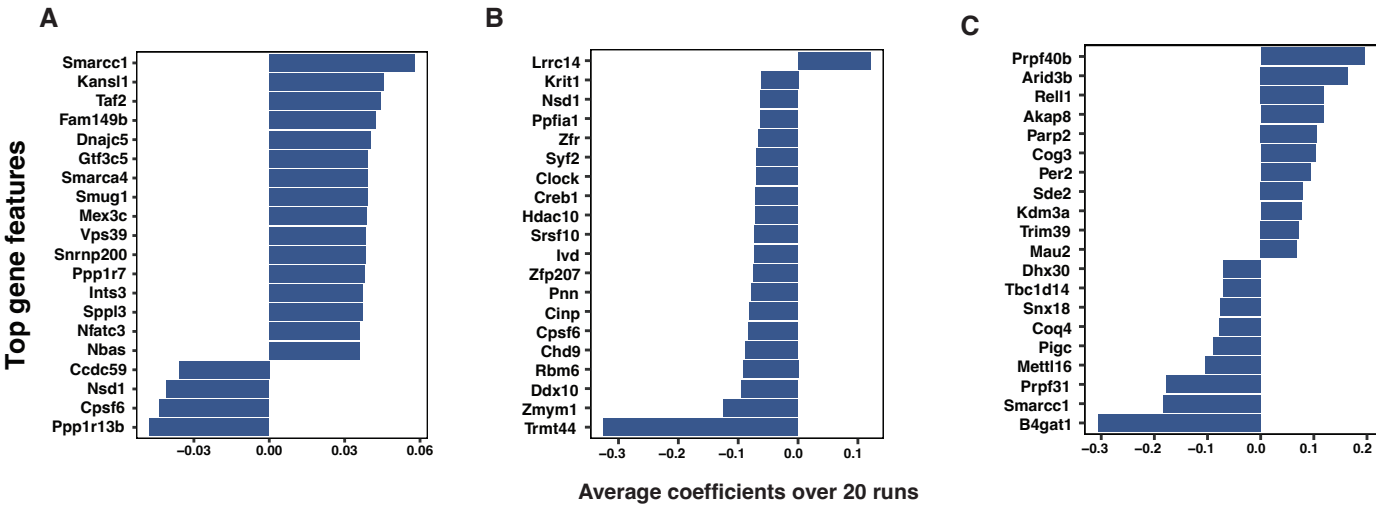

Supplement: Supplementary file 4 — Additional file 4: Gene features by machine learning approach. (A) Top 20 gene features predictive for sex differences in the old age group, (B) Top 20 gene features predictive for age group in the female samples, and (C) Top 20 gene features predictive for age group in the male samples. The gene features were selected by generalized linear regression with elastic net regularization through a 20-repeated 5-fold cross-validation approach. Top genes features were ranked by the absolute average coefficient over 20 runs. Gene features with positive coefficients were positively predictive for females or the old age group. [file 13293_2024_666_MOESM4_ESM.pdf]

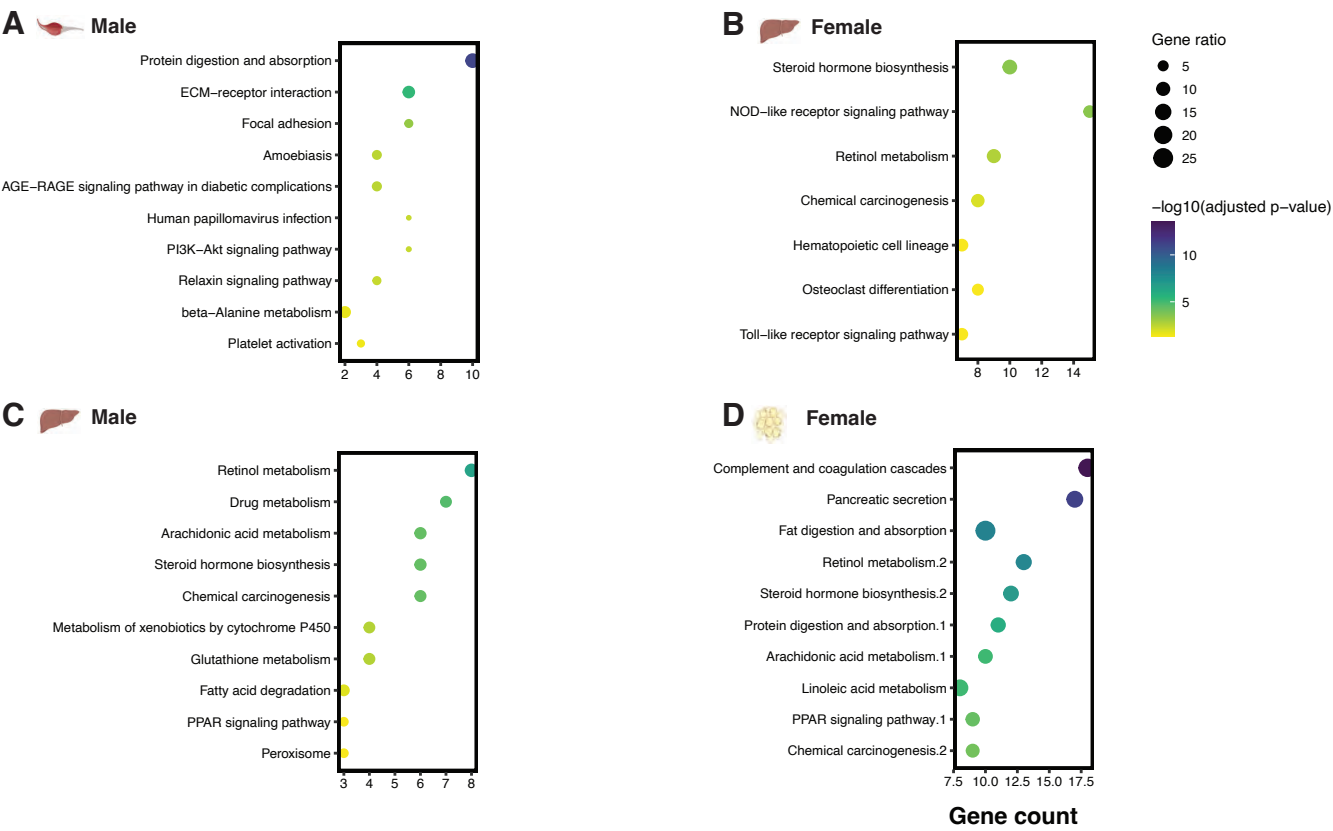

Supplement: Supplementary file 5 — Additional file 5: Over-represented pathways from tissue specific gene sets in the aging process. The gene sets were derived from the differentially expressed genes determined when comparing the old to the young age group in three types of tissues and from both sexes respectively. Sex specific DEGs that were not overlapped with the rest of the DEG lists were obtained, and those unique DEG lists containing greater than 30 genes were subjected to enrichment analysis respectively. Enriched pathways were detected in male specific genes in gastrocnemius muscle, female specific genes in Liver, male specific genes in Liver and female specific genes in white adipose tissues. [file 13293_2024_666_MOESM5_ESM.pdf]

## Additional file 6

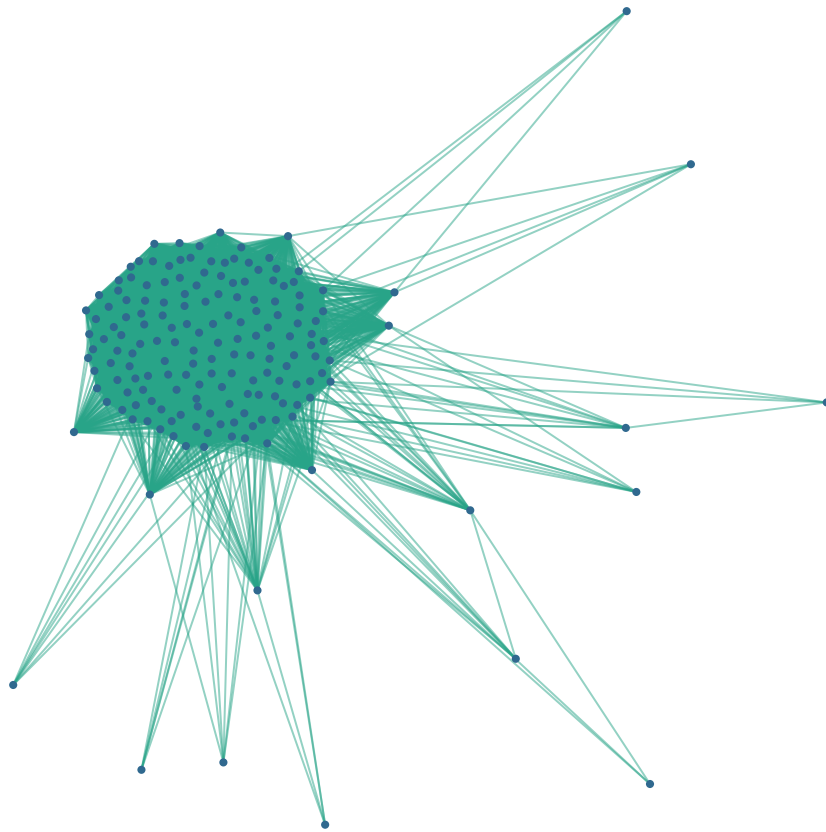

Supplement: Supplementary file 6 — Additional file 6: Co-expression network by weighted correlation network analysis. Gene expression data from all the 56 samples were subjected to co-expression network analysis. Network of the gene module that was determined associated with aging regardless of sex is shown. Nodes represent candidate hub genes. [file 13293_2024_666_MOESM6_ESM.pdf]
