# Supplementary material for: Sex dimorphism and tissue specificity of gene expression changes in aging mice
Source: Biol Sex Differ. 2024 Oct 31;15:89. doi: 10.1186/s13293-024-00666-4 (PMC11529319; doi:10.1186/s13293-024-00666-4)

Additional file 7

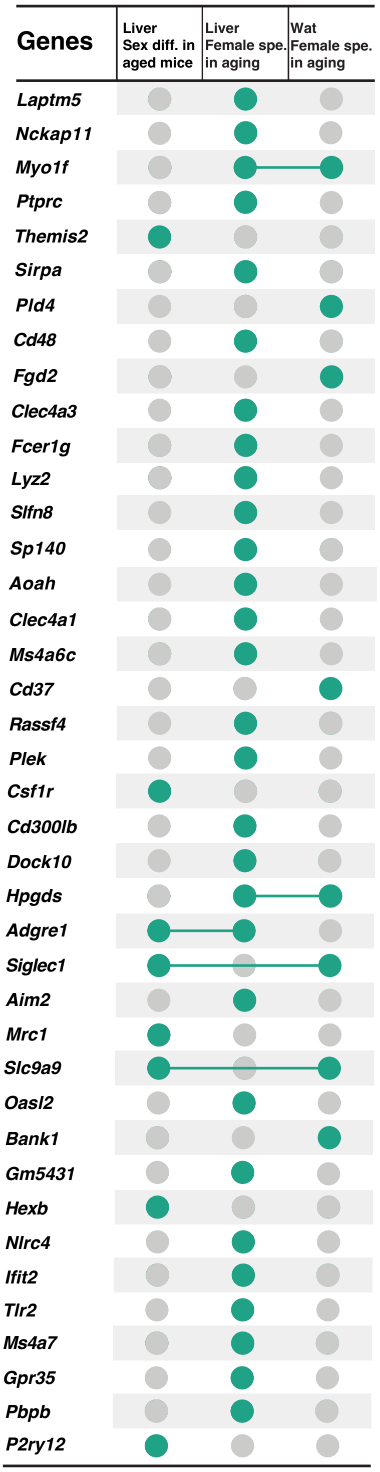

Supplement: Supplementary file 7 — Additional file 7: List of overlapping 40 hub genes from the co-expression network analysis. 40 hub genes were also identified in the previously selected differentially expressed genes from either the comparisons between female and male in the old age group or the comparisons between the old and the young age group of both sexes, in liver and white adipose tissue [file 13293_2024_666_MOESM7_ESM.pdf]
